# Pre-miRNA Hsa-Let-7a-2: a Novel Intracellular Partner of Angiotensin II Type 2 Receptor Negatively Regulating its Signals

**DOI:** 10.7150/ijbs.70455

**Published:** 2022-05-01

**Authors:** Xiaoyan Liu, Zhenzhen Chen, Shuangyue Li, Ling Jin, Xiao Cui, Changting Cui, Yue Deng, Qiannan Gao, Luyun Fan, Yaping Niu, Wenjie Wang, Chunmei Cui, Jiuchang Zhong, Qinghua Cui, Bin Geng, Jun Cai

**Affiliations:** 1 Department of Cardiology, Heart Center, Beijing Key Laboratory of Hypertension Research, Medical Research Center, Beijing Chao-Yang Hospital, Capital Medical University, Beijing 100020, China.; 2 Hypertension Center, Fuwai Hospital, Chinese Academy of Medical Sciences and Peking Union Medical College, State Key Laboratory of Cardiovascular Disease, National Center for Cardiovascular Diseases, Beijing 100037, China.; 3 Department of Biomedical Informatics, School of Basic Medical Sciences, Center for Non-Coding RNA Medicine, Third Hospital, Peking University, Beijing 100191, China.

**Keywords:** pre-miRNA, hsa-let-7a-2, AGTR2

## Abstract

G protein-coupled receptors (GPCRs) are the largest family of druggable targets, and their biological functions depend on different ligands and intracellular interactomes. Some microRNAs (miRNAs) bind as ligands to RNA-sensitive toll-like receptor 7 to regulate the inflammatory response, thereby contributing to the pathogenesis of cancer or neurodegeneration. It is unknown whether miRNAs bind to angiotensin II (Ang II) type 2 receptor (AGTR2), a critical protective GPCR in cardiovascular diseases, as ligands or intracellular interactomes. Here, screening for miRNAs that bind to AGTR2, we identified and confirmed that the pre-miRNA hsa-let-7a-2 non-competitively binds to the intracellular third loop of AGTR2. Functionally, intracellular hsa-let-7a-2 overexpression suppressed the Ang II-induced AGTR2 effects such as cAMP lowering, RhoA inhibition, and activation of Src homology 2 domain-containing protein-tyrosine phosphatase 1, whereas hsa-let-7a-2 knockdown enhanced these effects. Consistently, overexpressed hsa-let-7a-2 restrained the AGTR2-induced antiproliferation, antimigration, and proapoptosis of cells, and vasodilation of mesenteric arteries. Our findings demonstrated that hsa-let-7a-2 is a novel intracellular partner of AGTR2 that negatively regulates AGTR2-activated signals.

## Introduction

G protein-coupled receptors (GPCRs) are characterized by having seven transmembrane helices and six extracellular and intracellular loops and are the largest family of druggable targets 1. The activation of GPCRs translates the ligand's information into intracellular signals by a heterotrimeric G protein or β-arrestin-biased activation 2. Different ligands, such as hormones, photons, ions, proteins (chemokines), neurotransmitters, metabolites, and naturally produced synthetic agonists or antagonists determine the GPCRs' functions 1.

MicroRNAs (miRNAs) are 20-22-nt small non-coding RNAs that are first transcribed into relatively long primary miRNAs (pri-miRNAs), then processed by Drosha into 60-70-nt precursor miRNAs (pre-miRNAs) in the nucleus, and finally spliced by Dicer into mature miRNAs in the cytoplasm 3. Recently, miR-21 and miR-29a in exosomes were found to trigger inflammation by binding as ligands to toll-like receptor 7 (TLR7) and TLR8 in macrophages 4-5. Other miRNAs such as let-7b, miR-K-10b, and some virus miRNAs also function as “ligands”, binding to and activating protein receptors 6-8. Whether miRNAs can bind to other GPCRs and induce specific intracellular signals are still unknown.

The renin-angiotensin-aldosterone system plays an essential role in hypertension. There are two types of angiotensin II (Ang II) receptors (GPCR receptors): Ang II type 1 receptor (AGTR1) and AGTR2 9. AGTR1-dependent cardiovascular mechanisms have been well established, and selective blockers are the first-line drugs for hypertension. In contrast, AGTR2 activation induces different signaling and has opposite effects to those of AGTR1 and is beneficial in cardiovascular injury 10. Strikingly, AGTR2 has high affinity to COVID-19 spike protein, contributing to lung infection in coronavirus disease 2019 11.

The present study screened for miRNAs that bind to AGTR2 and investigated their interaction-induced functions and signals.

## Results

### Pre-miRNA hsa-let-7a-2 binds to AGTR2

**Figure 1:**
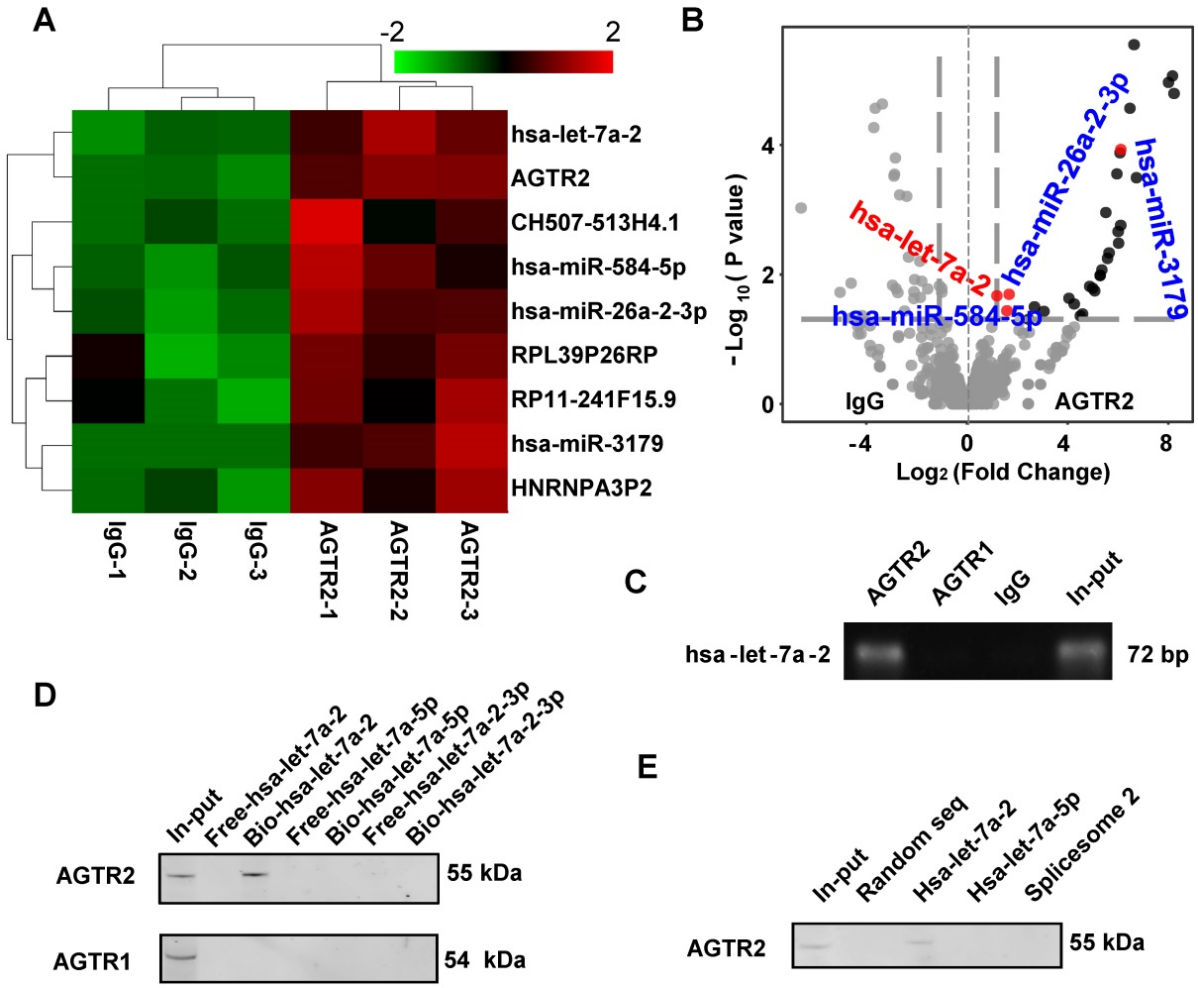
**Identification of hsa-let-7a-2 binding to AGTR2. (A)** Heatmap of AGTR2-binding RNAs including microRNAs and mRNAs compared with IgG by infrared-UV-C crosslinking immunoprecipitation assay (irCLIP) and RNA-sequencing. **(B)** Volcano plot showing the RNAs with changes in ATGR2 binding compared with IgG. **(C)** RNA-binding protein immunoprecipitation with anti-AGTR1 or anti-AGTR2 antibody; hsa-let-7a-2 was identified by RT-PCR.** (D)** Using chromatin isolation with RNA purification pull-down (ChRIP), AGTR2 was detected by a biotin-labelled DNA probe targeting hsa-let-7a-2 (Bio-hsa-let-7a-2) but not by probes unlabeled (Free-hsa-let-7a-2) or targeting hsa-let-7a-5p (Bio-hsa-let-7a-5p, Free-hsa-let-7a-5p) or hsa-let-7a-2-3p (Bio-hsa-let-7a-2-3p, Free-hsa-let-7a-2-3p). **(E)** Hsa-let-7a-2 binding to AGTR2 confirmed by RNA pull-down assay.

Using modified infrared-UV-C crosslinking immunoprecipitation (irCLIP) followed by RNA-sequencing, we identified four miRNAs that bound to AGTR2 (Figure 1A, B), excluding unspecific binding of IgG. Because AGTR2 is not a traditional RNA binding protein, we failed to find a conserved domain of these miRNAs. Next, using RNA-binding protein immunoprecipitation (RIP) followed by reverse transcription PCR (RT-PCR), we confirmed that four mature miRNAs (hsa-let-7a-2-3p, hsa-miR-26a-2-3p, hsa-miR-3179, and hsa-miR-584-5p), and one pre-miRNA hsa-let-7a-2 (Figure S1) bound to AGTR2. Given that let-7 is the most conserved miRNA that plays a critical role in growth and development and can activate TLR7 to induce neurodegeneration as well as being the only pre-miRNA that bound to AGTR2, we selected hsa-let-7a-2 as the candidate to investigate its biological functions upon interaction with AGTR2 8.

The RIP assay showed that hsa-let-7a-2 specifically bound to AGTR2, but not to AGTR1 (Figure 1C). By contrast, chromatin isolation RNA purification pull-down (ChIRP) (Figure 1D), RNA pull-down assay (Figure 1E), and microscale thermophoresis (MST) assay revealed that intact hsa-let-7a-2, but not its mature miRNAs hsa-let-7a-5p, hsa-let-7a-2-3p, or another spliceosome 2 (Figure S2), bound to AGTR2 but not to AGTR1 (Figure S3). Taken together, these data showed that the pre-miRNA hsa-let-7a-2 specifically bound to AGTR2.

### Hsa-let-7a-2 non-competitively binds to AGTR2

**Figure 2:**
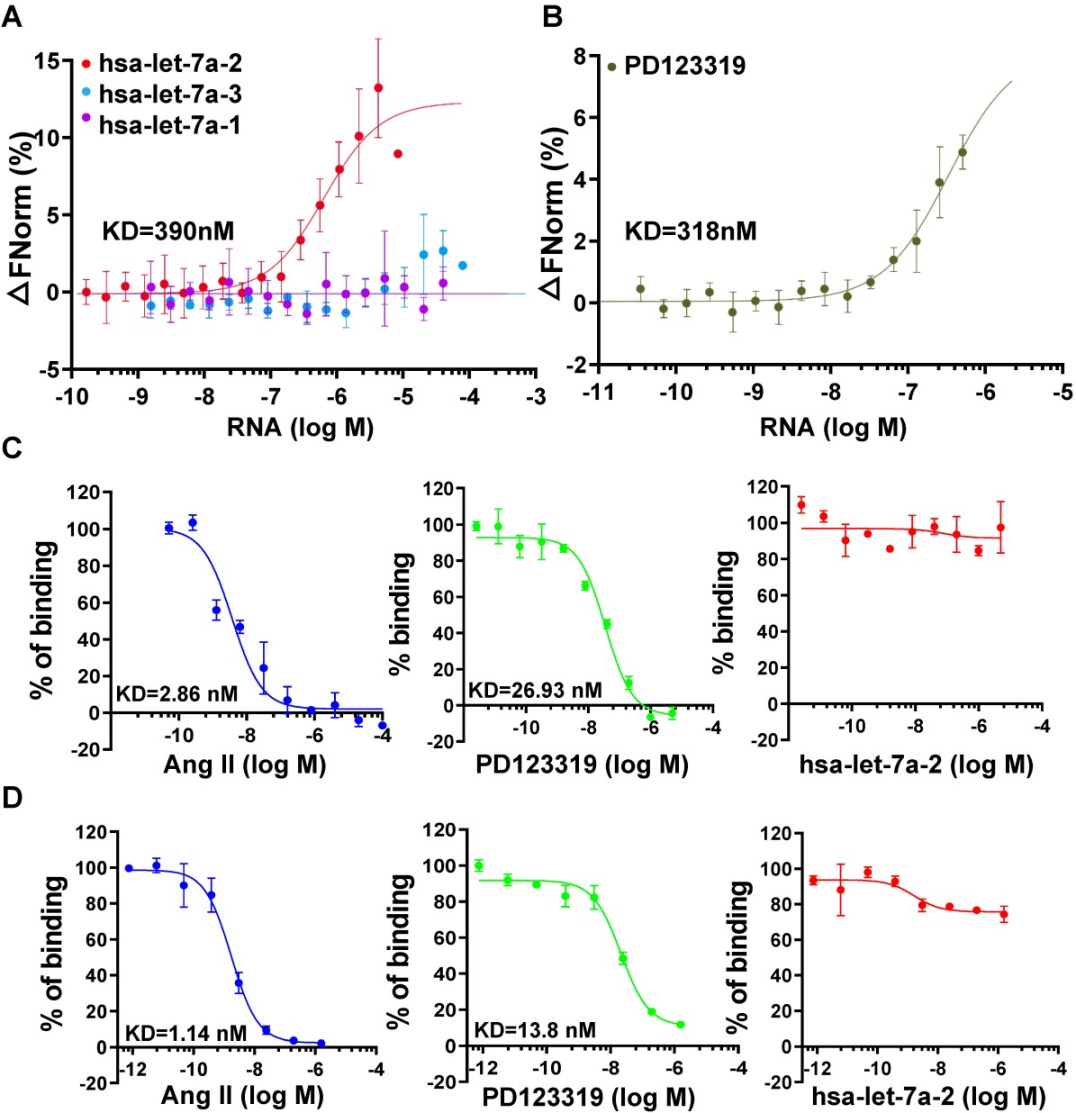
** Non-competitive binding of hsa-let-7a-2 with AGTR2. (A)** The kinetics of pre-miRNA binding to AGTR2 assayed by microscale thermophoresis (MST), with purified AGTR2-GFP protein as the receptor and hsa-let-7a-1, 2, and 3 as the ligands. **(B)** The binding kinetics of AGTR2 with its inhibitor, PD123319, as a positive control. **(C, D)** Competitive inhibition assay performed with [125-I]-sar1-Ile8-angiotensin II as a radioligand (C) or fluorescent ligand (D) to confirm the binding kinetics. Data are presented as means ± SD; n = 3 experiments.

To investigate the kinetic characteristics of hsa-let-7a-2 binding with AGTR2, the MST assay was used with purified AGTR2-GFP fusion protein as the receptor and *in vitro*-produced RNAs as the ligands. Intriguingly, hsa-let-7a-2, but not its homologs hsa-let-7a-1 and hsa-let-7a-3 (sequences are shown in Figure S4), dynamically bound to AGTR2 (Figure 2A) and had similar dissociation coefficient (KD) values as its inhibitor, PD123319 (Figure 2B). In contrast, hsa-let-7a-2 did not bind to boiled AGTR2 (negative control; Figure S5), further suggesting the specific binding of hsa-let-7a-2 with AGTR2. The competitive binding assay using the radioligand (Figure 2C) or fluorescent ligand assay (Figure 2D) demonstrated that hsa-let-7a-2 non-competitively bound to AGTR2 compared with the naïve ligand (Ang II). The KD value of PD123319 was almost 10 times that of Ang II. Because of the assay sensitivity, the KD value determined by the MST assay was larger than that determined by the radioligand or fluorescent ligand assay. Thus, hsa-let-7a-2 bound to AGTR2 at nanomole per liter concentrations.

**Figure 3:**
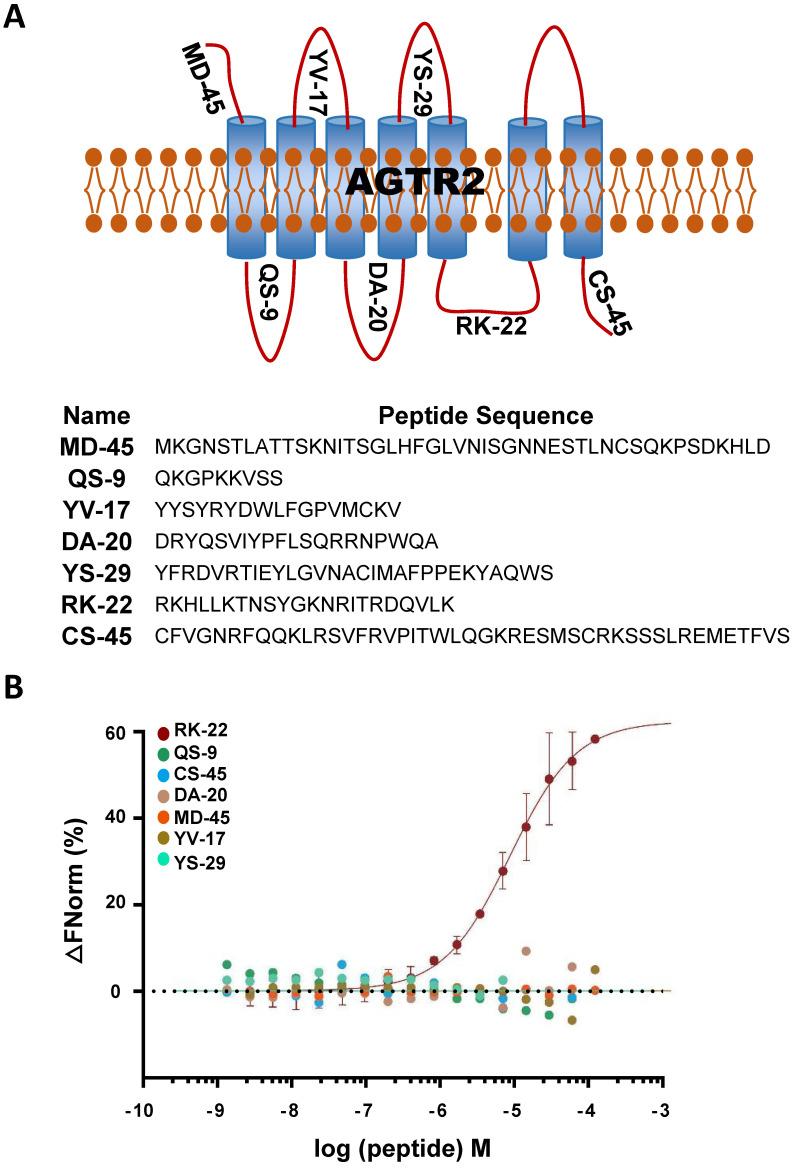
** hsa-let-7a-2 bound to the intracellular third loop of AGTR2. (A)** A schematic diagram of the extracellular and intracellular loops of AGTR2, including the names and sequences of the synthetic loop peptides. **(B)** hsa-let-7a-2 binding to the different loops of AGTR2 was examined by MST assay, with the synthetic loop peptides as the receptors and hsa-let-7a-2 as the ligand. Data are presented as means ± SD; n = 3 experiments.

Considering the non-competitive binding of hsa-let-7a-2 and AGTR2, there may be a different binding model of hsa-let-7a-2 with Ang II. As a GPCR, AGTR2 has seven transmembrane helices and six extracellular and intracellular loops. By determining the binding of hsa-let-7a-2 with synthesized loop peptides *in vitro* (Figure 3 A, B), we found that hsa-let-7a-2 specifically bound to RK22, the intracellular third loop of AGTR2 (Figure 3B), which is the same site for G_αi_ binding 12 and Src homology 2 domain-containing protein-tyrosine phosphatase 1 (SHP-1) activation 13. Co-immunoprecipitation results showed that, in HEK293 cells overexpressed with AGTR2 and G_αi-2_/ G_αi-3_, overexpression of hsa-let-7a-2 significantly decreased the interaction of G_αi-2_/ G_αi-3_ with AGTR2 (Figure S6), further suggesting that hsa-let-7a-2 competes with G_αi-2_/ G_αi-3_ in combination with AGTR2.

### Hsa-let-7a-2 negatively regulates AGTR2 post-receptor signaling pathways

As the interaction site of hsa-let-7a-2 with AGTR2, the intracellular third loop of AGTR2 is also an initiating site of post-receptor signals. cAMP inhibition, SHP-1 activation, and RhoA/Rho kinase (ROCK) activation are crucial AGTR2 post-receptor signaling pathways 14, 15, 16, 17. To investigate the effect of hsa-let-7a-2 on AGTR2-related intracellular signals, hsa-let-7a-1/2/3 or control plasmids were transfected into wild type (WT), Dicer^-/-^, Dicer^-/-^AGTR1^-/-^, and AGTR2^-/-^ human embryonic kidney 293 (HEK293) cells (Figure S7). In WT and AGTR2^-/-^ cells, transfection of hsa-let-7a-1/2/3 plasmids upregulated the expression of both the pre-miRNAs (hsa-let-7a-1/2/3) and the mature-miRNAs (hsa-let-7a-5p for hsa-let-7a-1/2/3, hsa-let-7a-2-3p for hsa-let-7a-2, and hsa-let-7a-3p for hsa-let-7a-1/3; Figure S8A-F). However, in Dicer^-/-^ and Dicer^-/-^AGTR1^-/-^ cells, transfection of these plasmids mainly upregulated the expression of the pre-miRNAs (hsa-let-7a-1/2/3; Figure S8A-F).

**Figure 4:**
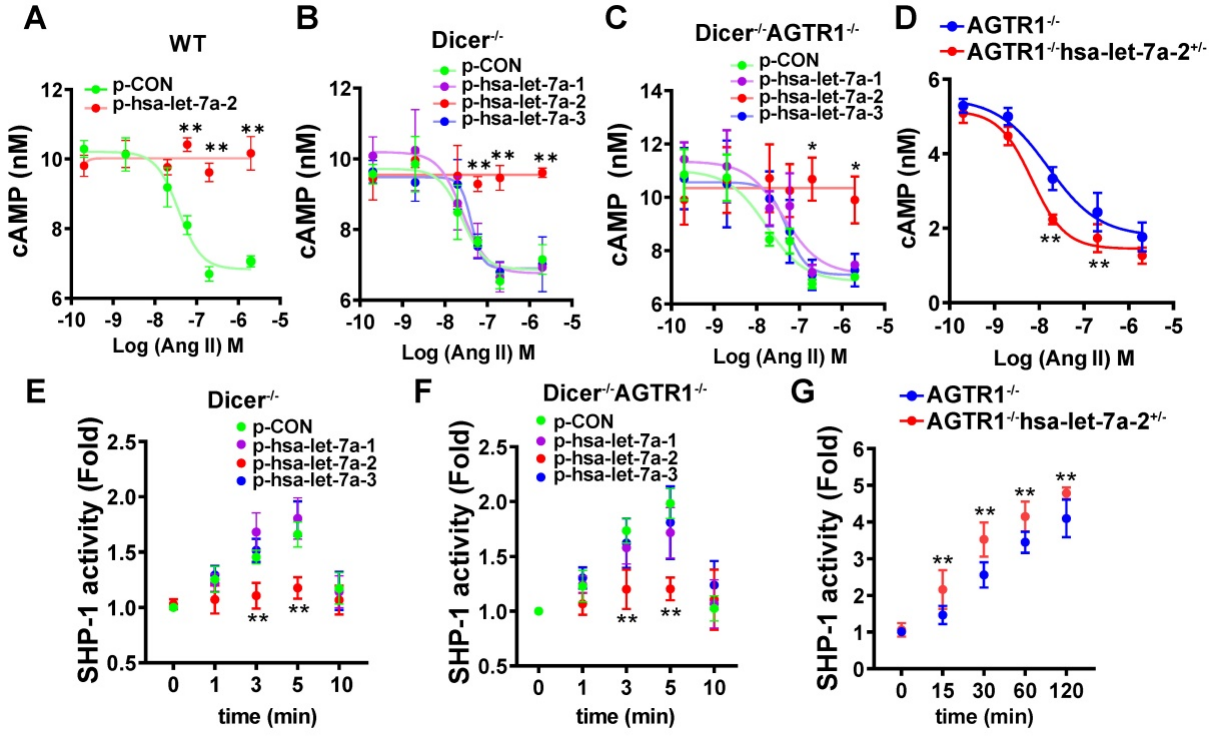
**Hsa-let-7a-2 negatively regulated Ang II-AGTR2 related cAMP and SHP-1 signals. (A)** Under AGTR1 inhibition with losartan and adenylate cyclase stimulation with forskolin, the intracellular cAMP level was examined after Ang II treatment in cells overexpressing hsa-let-7a-2 (p-hsa-let-7a-2) or control plasmid (p-CON). ** *P* < 0.01 vs. p-CON.** (B)** Under the same conditions, Ang II-induced cAMP alterations were examined in Dicer^-/-^ cells overexpressing hsa-let-7a-2 or hsa-let-7a-1/3. ** *P* < 0.01 vs p-CON. **(C)** Dicer^-/-^AGTR1^-/-^ double-knockout cells overexpressing hsa-let-7a-2 or hsa-let-7a-1/3 were pretreated with forskolin and the Ang II-induced cAMP level was measured. * *P* < 0.05 vs. p-CON.** (D)** AGTR1^-/-^hsa-let-7a-2^+/-^ cells and AGTR1^-/-^ cells were pretreated with forskolin and the Ang II-induced cAMP level was compared. ** *P* < 0.01 vs. AGTR1^-/-^. **(E)** Under AGTR1 inhibition with losartan, the Ang II-induced SHP-1 activity was examined in Dicer^-/-^ cells overexpressing hsa-let-7a-2 or hsa-let-7a-1/3. ** *P* < 0.01 vs. p-CON. **(F)** Effect of hsa-let-7a-2 or hsa-let-7a-1/3 on Ang II-induced SHP-1 activity in Dicer^-/-^AGTR1^-/-^ cells. ** *P* < 0.01 vs. p-CON. **(G)** Knockdown of hsa-let-7a-2^+/-^ enhanced SHP-1 activity in AGTR1^-/-^ cells. ** *P* < 0.01 vs. AGTR1^-/-^. n = 4-8 experiments.

HEK293 cells express both AGTR1 and AGTR2 receptors (Figure S9). Upon pre-blocking AGTR1 with losartan, Ang II lowered in a dose-dependent manner the forskolin (0.6 μM)-induced cAMP level, and this effect was reversed by hsa-let-7a-2 overexpression in WT cells (Figure 4A). To avoid the interference of mature miRNAs, we used Dicer^-/-^ HEK293 cells (Figure S7A). Consistently, hsa-let-7a-2, but not hsa-let-7a-1 or hsa-let-7a-3, blocked the decrease in cAMP level in Dicer^-/-^ cells (Figure 4B). Furthermore, only hsa-let-7a-2 had the same effect in Dicer^-/-^AGTR1^-/-^ cells (Figure S7B and Figure 4C). Conversely, hsa-let-7a-2 knockdown in AGTR1^-/-^ cells (AGTR1^-/-^hsa-let-7a-2^+/-^, Figure S7C) amplified the AGTR2-induced reduction in the cAMP level (Figure 4D). In contrast, Ang II did not change the cAMP level in AGTR2^-/-^ cells, whether hsa-let-7a-2 was overexpressed or not (Figure S7D and Figure S10A).

Consistently, hsa-let-7a-2 suppressed the Ang II-induced SHP-1 activity in Dicer^-/-^ cells (Figure 4E) and Dicer^-/-^AGTR1^-/-^ cells (Figure 4F), but not in AGTR2^-/-^ cells (Figure S10B). In contrast, the Ang II-induced SHP-1 activity was continuously increased in AGTR1^-/-^hsa-let-7a-2^+/-^ cells compared with that in AGTR1^-/-^ cells (Figure 4G).

**Figure 5:**
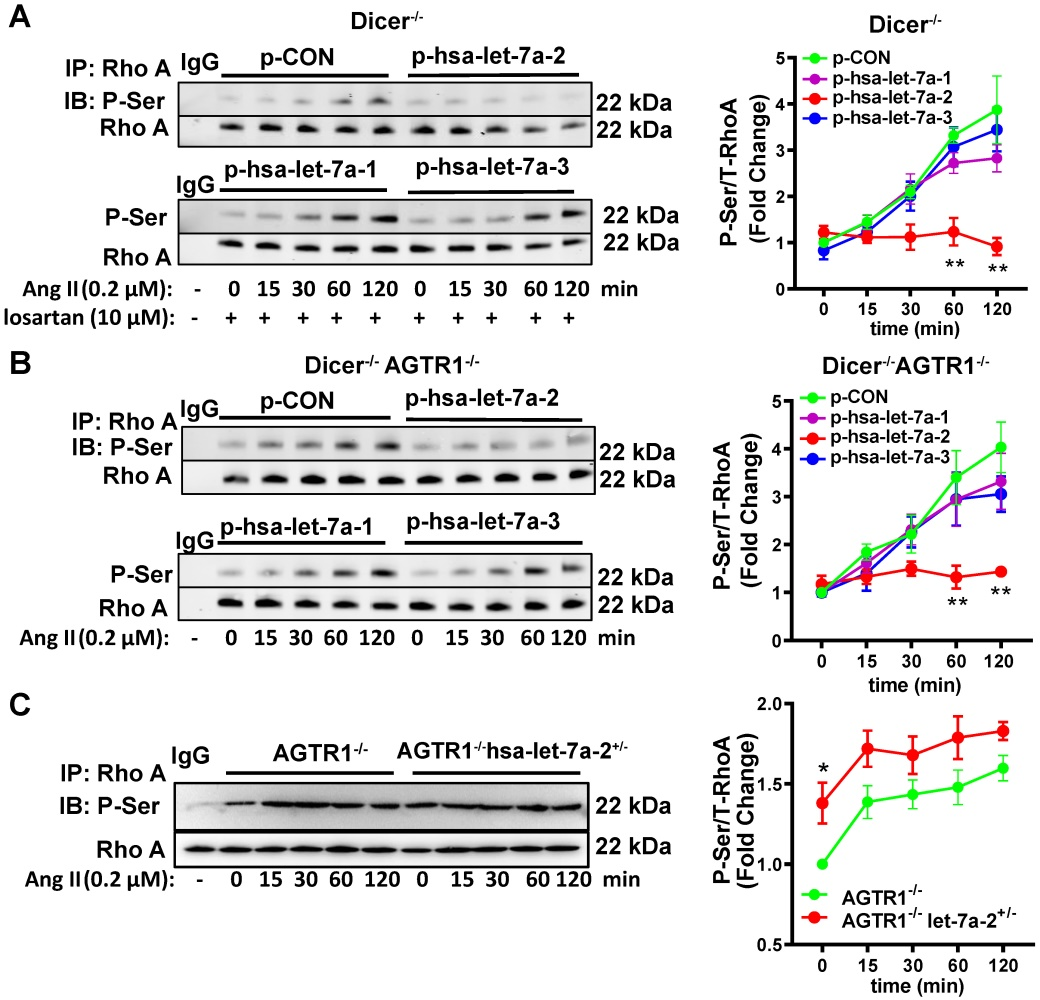
** Hsa-let-7a-2 negatively regulated Ang II-AGTR2 induced RhoA phosphorylation. (A)** Under AGTR1 inhibition, the effect of hsa-let-7a-2 or hsa-let-7a-1/3 overexpression on RhoA serine phosphorylation in Dicer^-/-^ cells was examined. ** *P* < 0.01 vs. p-CON. **(B)** RhoA serine phosphorylation was examined in Dicer^-/-^AGTR1^-/-^ cells overexpressing hsa-let-7a-2 or hsa-let-7a-1/3. ** *P* < 0.01 vs. p-CON. **(C)** The changes in Ang II-induced RhoA serine phosphorylation were examined in AGTR1^-/-^ hsa-let-7a-2^+/-^ cells. *P < 0.01 vs. AGTR1^-/-^. n = 4-8 experiments.

RhoA phosphorylation at Ser188 inhibits its activity, causing vasodilation 17-18. Similarly, Ang II induced RhoA phosphorylation at Ser188 in Dicer^-/-^ and Dicer^-/-^AGTR1^-/-^ cells in a time-dependent manner, whereas overexpression of hsa-let-7a-2, but not hsa-let7a-1 or 3, blocked this effect (Figure 5A-B). As expected, Ang II did not change the Ser188 phosphorylation level of RhoA in AGTR2^-/-^ cells whether hsa-let-7a-1/2/3 was overexpressed or not (Figure S10C). In contrast, hsa-let-7a-2 knockdown in AGTR1^-/-^ cells enhanced RhoA phosphorylation (Figure 5C).

Therefore, gain/loss-of-function studies targeting cAMP, SHP-1 activity, or RhoA phosphorylation as ATGR2-activated signals revealed that hsa-let-7a-2 binding to AGTR2 negatively regulated AGTR2 post-receptor signaling.

### Hsa-let-7a-2 overexpression negatively regulates AGTR2-induced cellular functions

**Figure 6:**
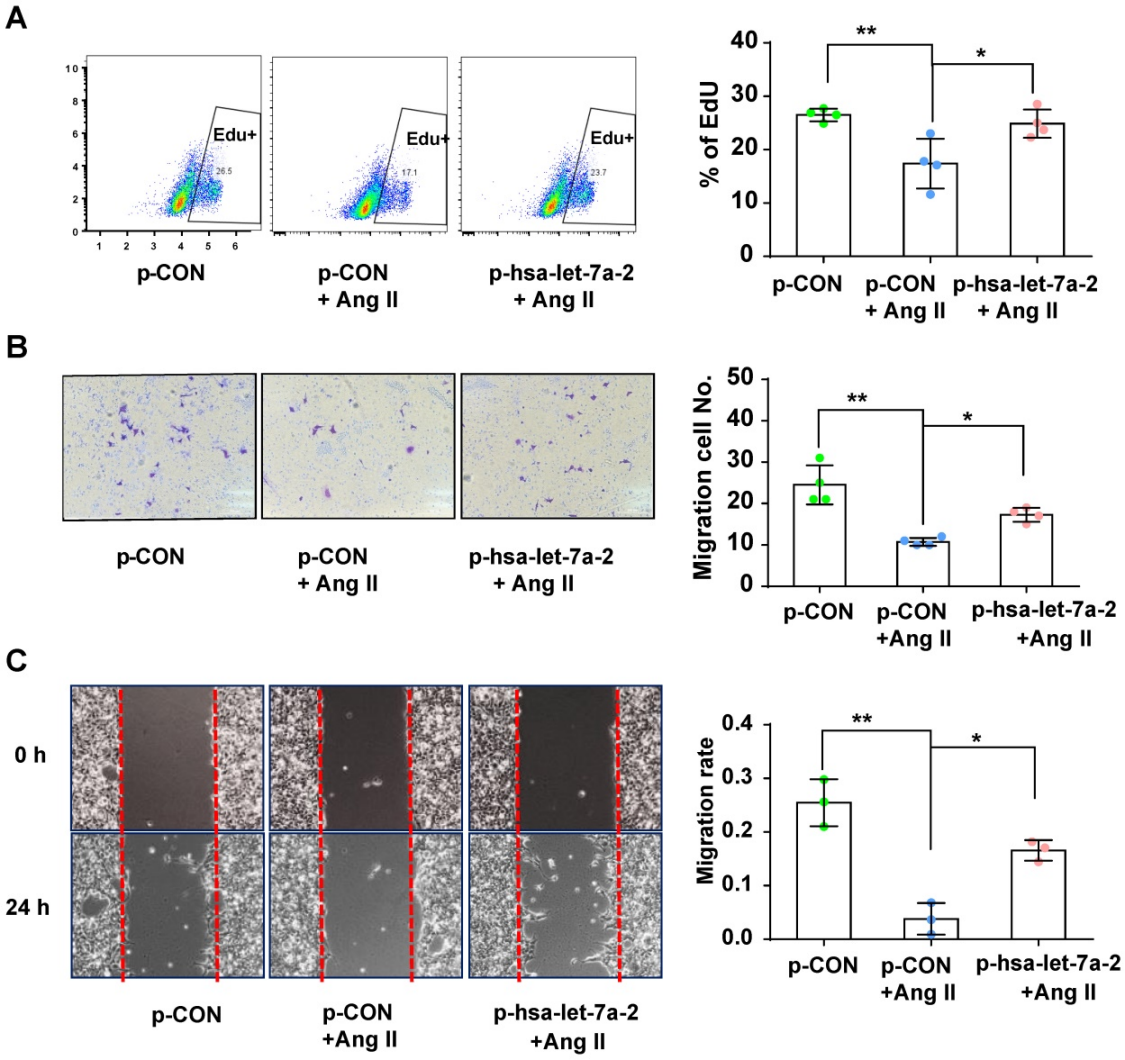
**Effect of hsa-let-7a-2 overexpression on cell proliferation and migration in Dicer^-/-^AGTR1^-/-^ cells. (A)** AngII-induced cell proliferation was analyzed by Edu incorporation assay with overexpression of hsa-let-7a-2 (p-hsa-let-7a-2) or control vector (p-CON). (B-C) AngII-induced cell migration was analyzed by transwell assay **(B)** or cell scratch assay **(C)** with overexpression of hsa-let-7a-2 (p-hsa-let-7a-2) or control vector (p-CON). Data are expressed as mean ± SD. *P<0.01, **P<0.01, n = 3-4 experiments.

**Figure 7:**
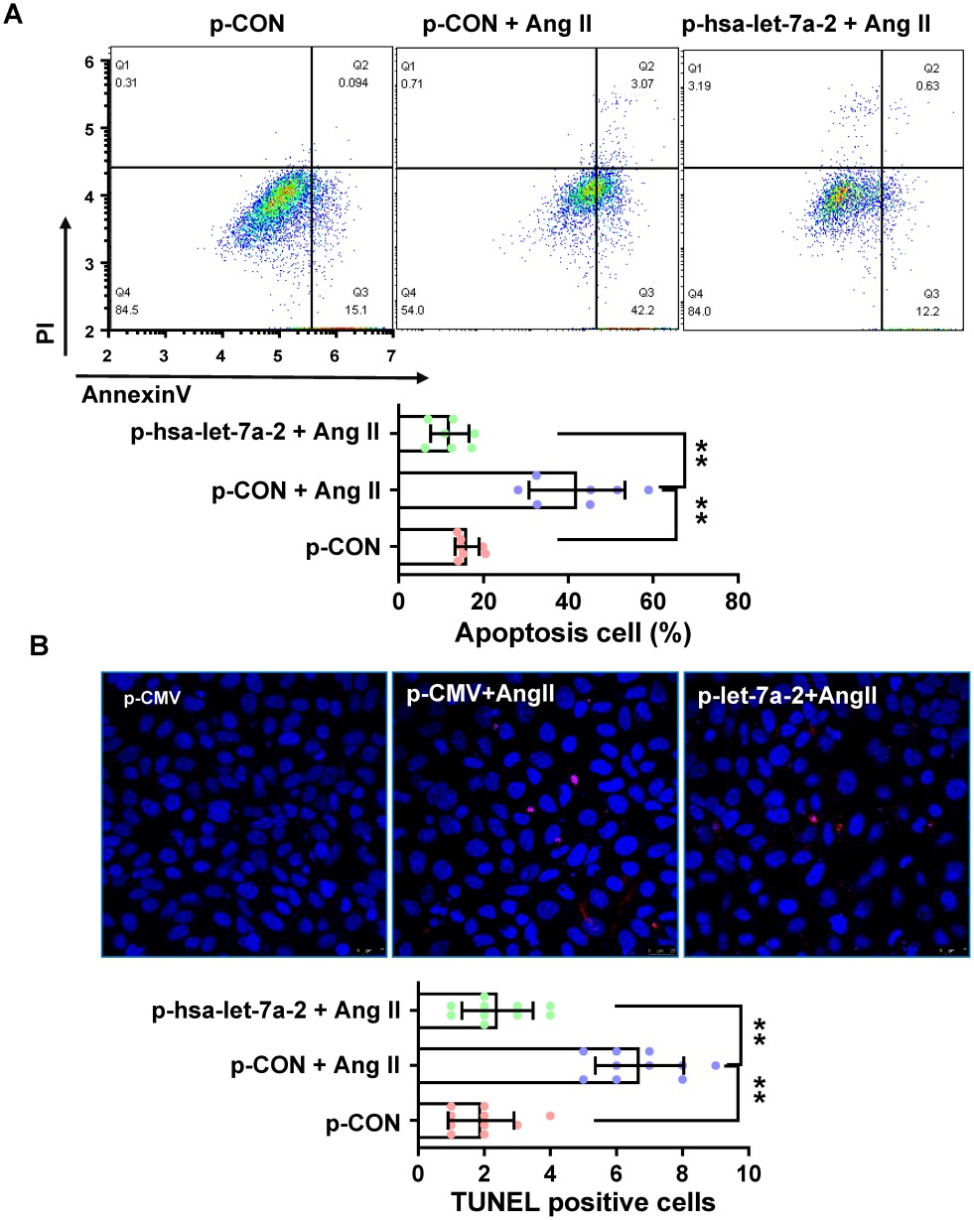
** Effect of hsa-let-7a-2 overexpression on cell apoptosis in Dicer^-/-^AGTR1^-/-^ cells.** (A) Ang II-induced cell apoptosis was analyzed by flow cytometry. (B) Cell apoptosis was measured by TUNEL assay. Pink color shows apoptotic cells. Data are expressed as mean ± SD. *P<0.01, **P<0.01, n = 6-10 experiments.

Previous reports have shown that AGTR2 activation inhibits endothelial migration and angiogenesis 19. Therefore, we assessed the effects of hsa-let-7a-2 overexpression on AGTR2-induced cellular functions in Dicer^-/-^AGTR1^-/-^ cells. Ang II reduced cell proliferation (Figure 6A) and migration (Figure 6B-C), which were reversed by hsa-let-7a-2 overexpression (Figure 6). Additionally, flow cytometry analysis and the terminal deoxynucleotidyl transferase dUTP nick end labeling (TUNEL) assay demonstrated that Ang II increased cell apoptosis, which was also reversed by hsa-let-7a-2 (Figure 7). Thus, hsa-let-7a-2 negatively regulated AGTR2-activation-dependent cellular functions.

### Hsa-let-7a-2 overexpression negatively regulates AGTR2-activated vasodilation and signaling

**Figure 8:**
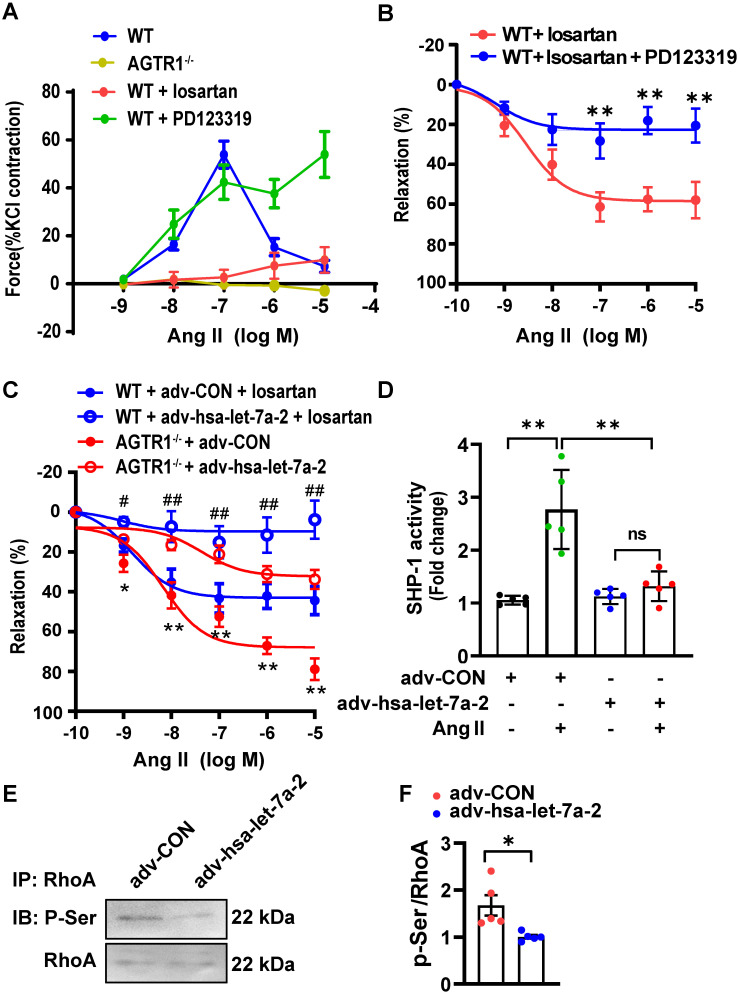
** hsa-let-7a-2 reduced AGTR2-activated vasodilation and signaling. (A)** The Ang II-induced class II mesenteric artery tension response in wild type (WT) mice pretreated with losartan (AGTR1 inhibitor) or PD123319 (AGTR2 inhibitor) and in AGTR1 knockout (AGTR1^-/-^) mice. **(B)** Negative regulation of AGTR2-activated mesenteric artery dilation under PD123319 treatment. ** *P* < 0.01 vs. WT + losartan. **(C)** Overexpression of hsa-let-7a-2 by adenovirus (adv-hsa-let-7a-2) regulated AGTR2-activated vasodilation in WT and AGTR1^-/-^ mice. GFP-adenovirus transfection was used as the control (adv-CON). * *P* < 0.05, ** *P* < 0.01 vs. AGTR1^-/-^ + adv-hsa-let-7a-2; # *P* < 0.05, ## *P* < 0.01 vs. WT + adv-CON + losartan. **(D)** hsa-let-7a-2 overexpression suppressed arterial tissue SHP-1 activity. ** *P* < 0.01, ^ns^ *P* > 0.05. **(E, F)** Effect of hsa-let-7a-2 on RhoA phosphorylation at Ser188 in AGTR1^-/-^ mesenterial artery. Data are presented as mean ± SD. * *P* < 0.05. n = 5-16 experiments.

AGTR2 activation has opposite effects to those of AGTR1 9. In WT mouse mesenteric arteries, Ang II induced quick constriction and then relaxation; however, the constriction was sustained by pretreatment with PD123319 (AGTR2 antagonist) but blocked by losartan treatment or AGTR1 deletion (Figure 8A). Furthermore, under AGTR1 inhibition with losartan, Ang II relaxed the phenylephrine pre-constricted arteries in a dose-dependent manner, similar to rat arteries 20, whereas this effect was blunted under AGTR2 inhibition (Figure 8B). These results confirmed the vasodilation upon AGTR2 activation and vasoconstriction upon AGTR1 activation. Using this model, we demonstrated that in WT mouse mesenteric arteries treated with losartan or in AGTR1^-/-^ mouse mesenteric arteries, overexpression of hsa-let-7a-2 by adenoviruses reduced ATGR2-dependent vasodilation compared with the control (Figure 8C), along with suppression of SHP-1 activity (Figure 8D) and RhoA phosphorylation at Ser188 (Figure 8E-F), which are tightly linked with vasodilation 18. These *ex vivo* experiments demonstrated that hsa-let-7a-2 negatively modulated AGTR2 functions and signaling pathways.

## Discussion

**Figure 9:**
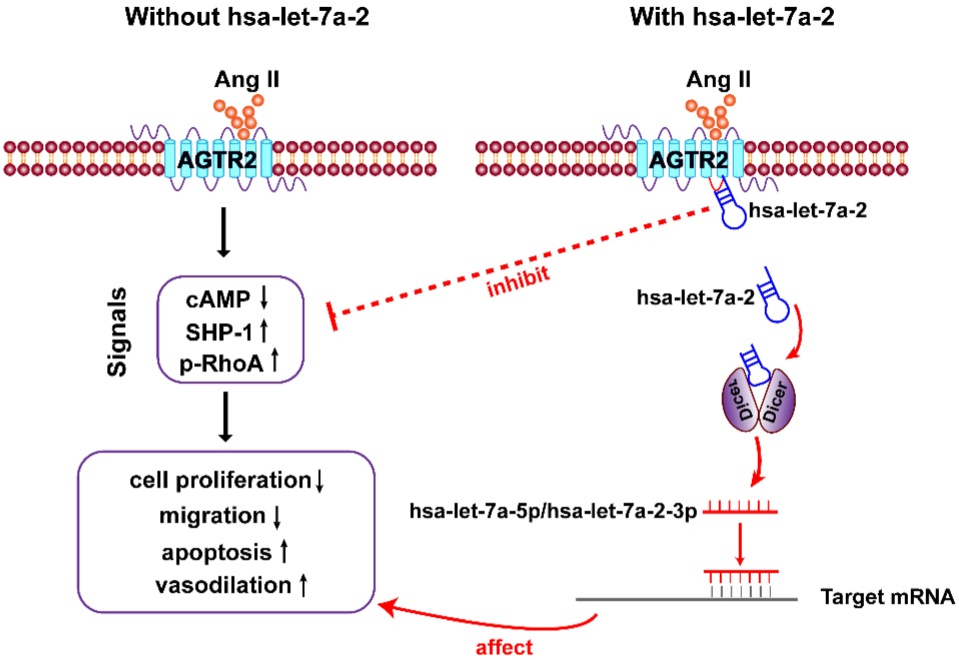
**Diagram of findings**. Hsa-let-7a-2 not only serves as the pre-miRNA of hsa-let-7a-5p and hsa-let-7a-2-3p, but also binds to the intracellular third loop of AGTR2 and negatively regulates AGTR2 signals and the related biological processes.

In the present study, we first clarified that in addition to being the pre-miRNA of hsa-let-7a-5p and hsa-let-7a-2-3p, hsa-let-7a-2 has important biological functions. As a nucleic acid molecule, hsa-let-7a-2 shares the same intracellular binding site as Gi proteins, and it negatively regulates AGTR2 signals and the related biological processes (Figure 9).

Let-7 miRNAs are a conserved miRNA family in animal species including humans. Functionally, let-7 miRNAs act as regulators of gene expression and play an essential role in development and tumor suppression 21. In humans, the precursors of human let-7a-1, let-7a-2, and let-7a-3 are encoded on chromosomes 9, 11, and 12, respectively; however, the production of their mature form is the same as for hsa-let-7a-5p 21. Here, we demonstrated that hsa-let-7a-2, but not hsa-let-7a-1 or 3, bound to AGTR2, and that the mature hsa-let-7a-5p did not bind to it. Because of the 15-25-bp difference among the sequences of hsa-let-7a-1, 2, and 3, we also measured the binding of mature hsa-let-7a-2-3p and spliceosome 2 (removing hsa-let-7a-5p) to AGTR2 and confirmed that these short RNAs did not bind. Furthermore, screening the four AGTR2-binding miRNAs, we did not find a conserved domain. Thus, the binding of hsa-let-7a-2 with AGTR2 is not sequence specific and may be structure specific.

The Croce group first identified tumor-secreted miR-21 and miR-29a as ligands that bind to TLR7 and TLR8 of immune cells, triggering a pro-metastatic inflammatory response and changing the microenvironment of tumor growth or metastasis 4. Extracellular let-7b has also been reported to activate TLR7 in neurons to trigger inflammation, thereby promoting neurodegeneration 8. Subsequent studies have also demonstrated that many miRNAs (animal or viral), as ligands, activated TLR7 to participate in the genesis of diseases 6, and this research also indicated that extracellular miRNAs act as a “hormone” to mediate a receptor's function. BecauseTLR7 and TLR8 are RNA-sensing receptors; whether miRNAs also activate or inhibit GPCRs (the largest receptor family) remains unclear. Here, we identified that hsa-let-7a-2 (a precursor of let-7a) can specifically bind to the intracellular third loop of the AGTR2 receptor.

AGTR2 has the characteristic motifs and signature residues of a GPCR, but it does not exhibit most of the classical post-receptor signaling 22. Because AGTR2 activation has vasodilator, natriuretic, antigrowth, antiproliferative, and proapoptotic effects and plays protective roles in cardiovascular injury 10, we investigated the role of hsa-let-7a-2 in AGTR2-activated signals and cellular functions. It has been reported that AGTR2 activation induced natriuresis and Na^+^-K^+^ ATPase inhibition in the inner cortex partly via cAMP 14, 23-24. AGTR2 activation also promoted SHP-1 activity by G protein 15, 25 or AT2 receptor interaction protein 26, and RhoA phosphorylation at Ser188 17-18 to regulate vasodilation. Here, we found that Ang II activation of AGTR2 decreased the forskolin-induced reduction in the cAMP level and elevated SHP-1 activity and RhoA phosphorylation under pharmacological inhibition or knockout of AGTR1. This inhibitory effect was abolished by hsa-let-7a-2, but not by hsa-let-7a-1 or 3. Consistent with the inhibition of AGTR2 signals, hsa-let-7a-2 binding to the third loop of AGTR2 reduced AGTR2 induced antiproliferation 12, antimigration 19, and proapoptosis 13 of cells, and vasodilation of mesenteric arteries 18. Thus, our study sheds new light on the pre-miRNA hsa-let-7a-2 interaction with and negative regulation of AGTR2 signals and biological functions.

The post-receptor signals of AGTR2 are complicated owing to the different G-proteins and other interaction proteins in different cells. Gi proteins such as Giα2, Giα3 27-28, and Gαs 15, which bind to the intracellular third loop 12, 29, are necessary for AGTR2 signals. The EGF receptor ErbB3 acts as a partner of AGTR2 and binds to the intracellular third loop, reducing the activity of tyrosine kinases such as insulin receptor 30-31. ATIP family proteins or promyelocytic zinc finger protein that bind to the C-terminal of AGTR2 participate in AGTR2 signaling 26, 32-33. These partners also contribute to the functional diversity of AGTR2 activation. Here, we identified a novel nucleic acid partner of AGTR2, hsa-let-7a-2, which interacted with the intracellular third loop and suppressed AGTR2 related signals and biological functions. AGTR2 receptor is predominantly expressed in embryonic, fetal, and neonatal tissues and regulates stem cell homeostasis 31, 34-35. Therefore, the AGTR2-let-7a-2 interaction is a novel intracellular signaling pathway.

In summary, our findings shed new light on hsa-let-7a-2, which bound to the intracellular third loop of AGTR2 and antagonized AGTR2-activated intracellular signals, cellular functions, and vasodilation. This study gives a novel working model of a pre-miRNA, which not only acts as the pre-miRNA of mature miRNAs, but also has important biological functions.

## Materials and Methods

### Generation of single and double-knockout cell lines

Single or double-knockout cell lines were generated by the CRISPR-Cas9 system 36. In brief, eight single guide RNAs (sgRNAs) targeting *DICER*, two sgRNAs targeting *AGTR1*, and two sgRNAs targeting *AGTR2* were designed and cloned into the lenti-CRISPR v2 plasmid (Cat. 52961, Addgene). To generate homozygous DICER, AGTR1, or AGTR2-knockout cell lines, the corresponding sgRNAs for each gene were transfected into HEK293 cells, which were continuously cultured for 48 h. Puromycin (2 μg/mL) was added to screen for positive expression cells. After low-density seeding, single clones were expanded in 96-well plates. PCR amplification and sequencing were performed to verify DNA knockout. Dicer^-/-^AGTR1^-/-^ cells were constructed by co-transfecting the plasmids containing the sgRNAs of *AGTR1* and *DICER*, and AGTR1+hsa-let-7a-2 double-knockout cells (AGTR1^-/-^hsa-let-7a-2^+/-^) were constructed by co-transfecting the plasmids containing the sgRNAs of *MIRLET7A2* and *DICER*, followed by screening with puromycin and single clone expansion. The sequences of the sgRNAs for *AGTR1*, *AGTR2*, *DICER*, and *MIRLET7A2* are shown in Table S1.

### Cell culture and plasmid transfection

HEK293, Dicer^-/-^, AGTR2^-/-^, AGTR1^-/-^, Dicer^-/-^AGTR1^-/-^, and AGTR1^-/-^hsa-let-7a-2^+/-^ HEK293 cells were cultured in Dulbecco's Modified Eagle Medium (DMEM) with 10% fetal bovine serum (FBS, Gibco BRL, Canada) and 100 units/mL penicillin and streptomycin (Invitrogen, Carlsbad, CA, USA). Cells were then incubated at 37°C in a humidified 5% CO_2_ atmosphere.

pCMV-MIRLET7A1 (Cat. SC400001), pCMV-MIRLET7A2 (Cat. SC400002), pCMV-MIRLET7A3 (Cat. SC400003), pCMV6-AGTR2 (Cat. RC223317), and pCMV6-AGTR1 (Cat. RC209773) were from Origene (Rockville, MD, USA). AGTR2-GFP and AGTR1-GFP fusion plasmids were constructed from the above plasmids. Transfections and co-transfections of plasmids into HEK293 cells were performed with Lipofectamine 3000 reagent (Invitrogen, Carlsbad, CA, USA) according to the manufacturer's instructions.

### Infrared cross-linking immunoprecipitation (irCLIP) and RNA-sequencing

The L3-azide-biotin substrate oligonucleotide for irCLIP (5′-OH-AGATCGGAAGAGCGGTTCAGAAAAAAAAAAAA/iAzideN/AAAAAAAAAAAA/3Bio/-3′) was synthesized by Integrated DNA Technologies (IDT). The final preA-L3-IR800-biotin DNA adaptor was constructed and purified as previously described 37. In brief, the cells in 15-cm dishes were quickly rinsed with ice-cold phosphate-buffered saline (PBS), aspirated on plate, cross-linked on ice with 254 nM UV-C at 0.3 J/cm^2^, incubated with ice-cold PBS/10 mM EDTA for 5 min, and then collected by scraping. Pelleted cells were lysed in SDS lysis buffer (1% SDS, 50 mM Tris, pH 7.5, 1 mM EDTA) and soluble fractions were mixed with two volumes of IP Dilution Buffer (1.1% Triton X-100, 50 mM Tris, pH 7.5, 1 mM EDTA, 450 mM NaCl). AGTR2 antibody (Abcam, Cambridge, MA, Cat. #ab92445) or IgG as a negative control pre-conjugated to Protein G Dynabeads (Thermo Fisher Scientific, Waltham, MA, USA, Cat. #10004D) were added to the lysate and rotated end-over-end for 1.5 h at 4°C. Immunoprecipitants were washed sequentially for 10 min each at 4°C with 1 mL of high-stringency buffer (20 mM Tris, pH 7.5, 120 mM NaCl, 25 mM KCl, 5 mM EDTA, 1% Trition-X100, 1% Na-deoxycholate), 1 mL of high-salt buffer (20 mM Tris, pH 7.5, 1 M NaCl, 5 mM EDTA, 1% Trition-X100, 1% Na-deoxycholate, 0.001% SDS), and 1 mL of low-salt buffer (20 mM Tris, pH 7.5, 5 mM EDTA). Beads were rinsed twice on magnet with 0.25 mL of NT2 buffer, and then with 0.1 mL of NT2 buffer (50 mM Tris, pH 7.5, 150 mM NaCl, 1 mM MgCl2, 0.0005% Igepal).

RNAse A (Affymetrix #70194Z) was diluted in NT2 buffer at a final concentration of 0.4 U/μL. The RNAse A digestion reactions had a total aqueous volume of 30 μL containing 6 μL of PEG400 (16.7% final). The reactions were chilled on ice before resuspension of the immunoprecipitants. The reactions were incubated at 30°C for 15 min in an Eppendorf Thermomixer, followed by centrifugation for 15 s at 1,400 rpm and a 90-s rest. Digestions were stopped by the addition of 0.5 mL of ice-cold high-stringency buffer. The immunoprecipitants were then quickly rinsed with 0.25 mL and 0.05 mL of ice-cold NT2 buffer. The digested complexes were then dephosphorylated with T4 PNK (NEB, Cat. M0210) for 30 min in an Eppendorf Thermomixer at 37°C, followed by centrifugation for 15 s at 1,400 rpm and a 90-s rest in a 30-μL reaction (50 mM Tris, pH 7.0, 10 mM MgCl2, 5 mM DTT) containing 10 units of T4 PNK, 0.1 μL of SUPERase-IN (Thermo Fisher Scientific, #AM2694), and 6 μL of PEG400. Dephosphorylation reactions were removed and immunoprecipitants were 3′-end-ligated with T4 RNA Ligase 1 (NEB, Cat. #M0204) overnight in an Eppendorf Thermomixer at 16°C, followed by centrifugation for 15 s at 1,400 rpm and a 90-s rest in a 30-μL reaction containing 10 units of T4 RNA Ligase 1, 1 pmole preA-DNA-adaptor, and 0.1 μL of SUPERase-IN. The 30-μL ligation reactions were supplemented with 6 μL of PEG400 (16.7% final).

On the following day, beads were placed on a magnetic stand, ligation reactions were removed, and beads were resuspended in 10 μL of 1× LDS sample buffer + reducing agent and heated for 15 min at 75°C. Samples were stored at -20°C or immediately resolved by SDS-PAGE with NuPAGE 4%-12% Bis-Tris Gels (1.0 mm × 12 well) at 180 V for 45 min. Resolved RNP complexes were wet-transferred to a nitrocellulose membrane at 400 mA for 60 min at 4°C. The nitrocellulose membrane was cut, and then digested with proteinase K. The recovered RNA was purified, and then sequencing and data analysis were performed by LC sciences (Hangzhou, China). Quantification of RNAs bound to AGTR2 was done based on number of reads per million (RPM) using the following formula:



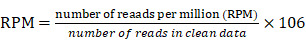



Compared with IgG group, |log 2 (fold change) |>1 and p< 0.05 were set as threshold cutoff of differentially detected in AGTR2 group.

### RNA-binding protein immunoprecipitation (RIP)

RIP was performed with the EZ-Magna RIP RNA-Binding Protein Immunoprecipitation Kit (Cat. #17-701, Millipore, CA, USA) according to the manufacturer's guidelines. Briefly, after transfection of HEK293 cells with hsa-let-7a-2 plasmid and AGTR2/AGTR1 plasmid for 24 hours, the cells were homogenized with RIP lysis buffer. Then, the cell lysates were incubated with anti-AGTR2 antibody, anti-AGTR1 antibody, or isotype‐matched IgG (5 μg of total antibody per immunoprecipitation) immobilized on magnetic beads at 4°C overnight. After extensive washing, the beads were digested with proteinase K (30 min at 55°C), the RNA was harvested by canonical phenol chloroform isoamyl extraction, and then precipitated with ethanol. The eluted RNA was reverse-transcribed into cDNA with a Reverse Transcription Kit (Promega, Fitchburg, USA), and hsa-let-7a-2 was analyzed by PCR with the primer sequences 5′-AGGTTGAGGTAGTAGGTTGTATAGT-3′ and 5′-GGAGGCTGTACAGTTATCTCCC-3′.

### Chromatin isolation by RNA purification (ChIRP)

ChIRP was performed as previously described with minor modifications 38. In brief, HEK293 cells co-transfected with AGTR1/AGTR2 and hsa-let-7a-2 plasmids in 10-cm cell culture dishes were irradiated by UV light for 2 min with lids off and PBS aspirated, then they were incubated in 3% formaldehyde for 10 min for chemical cross-linking. Cross-linked cells were homogenized with NP40 lysis buffer (20 mM pH 7.4 Tris, 1M NaCl, 1 mM EDTA, 1% NP40) and divided into equal amounts, and then 400 pmol of biotinylated antisense oligo pools or their free antisense oligo pools targeting hsa-let-7a-2 were added and incubated at 94°C for 1 min to open the stem loop of hsa-let-7a-2. After slowly cooling to room temperature, 100 μL of Streptavidin Magnet Spheres (Beaver for Life Science, Suzhou, China) were added to each sample to hybridize overnight at 4°C. Finally, the bound RNA and protein complexes were eluted by 1×protein loading buffer at 70°C for 10 min and analyzed by immunoblotting. The probe sequences were: probe-1, 5'-AGGAAAGCTAGGAGGCTGTACA-3', probe-2, 5'-TATCTCCCTTGATGTAATTCTA-3', probe-3, 5'-CTATACAACCTACTACCTCAAC-3'.

### RNA pull-down assay

The RNA pull-down assay was performed with a Pierce Magnetic RNA-Protein Pull-Down Kit (Pierce Biotechnology, Rockford, IL, USA) according to the manufacturer's specifications. Briefly, biotin-labeled hsa-let-7a-2, hsa-let-7a-5p, hsa-let-7a-2-3p, and a 72-nt random RNA sequence with the same composition of ribonucleotides as hsa-let-7a-2 were synthesized by Dharmacon. HEK293 cells were transfected with AGTR2 plasmid for 24 h before being lysed with lysis buffer. Cell lysates were incubated with streptavidin magnetic beads bound to biotin-labeled RNA at 4°C for 30 min with rotation. After extensive washing, the RNA-bound protein complexes were eluted by heating at 70°C for 10 min in 1×loading buffer. The AGTR2 protein present in the pull-down material was examined by western blot analysis.

### Radioligand receptor binding assay

Membrane proteins from hAGTR1 and hAGTR2 stable HEK293 cell lines were extracted and quantified by BCA. The total reaction volume was 150 μL, consisting of 120 μL membrane protein (5 μg of protein/well), 15 μL of [125-I]-sar1-Ile8-Ang II, and 15 μL of different concentrations of unlabeled competitor (hsa-let-7a-2, hsa-let-7a-5p, or hsa-let-7a-2-3p). After a 2-h incubation at room temperature, the reaction was stopped by rapid filtration through Unifilter GF/C plates, and then the plate was washed twice, dried at room temperature overnight, and finally read by TopCount.

### Fluorescent ligand binding assay

Fluorescent ligand binding assay was performed with a Tag-Lite Angiotensin AT2 Receptor Ligand Binding Assay kit (Cisbio) according to the manufacturer's protocol 39. Different concentrations of hsa-let-7a-2 (5 μL) were titrated into a solution containing a fixed concentration of fluorescent Ang II (3 nM, 5 μL) and a fixed amount of Tag-lite AGTR2-labeled cells (10 μL). After incubation at room temperature for 1 h, fluorescent signals were detected by a Ruby star fluorescent reader (BMG Labtech, Offenburg, Germany) at 665 and 620 nm. Homogeneous Time Resolved Fluorescence (HTRF) ratios were obtained by dividing the acceptor signal (665 nm) by the donor signal (620 nm) and multiplying this value by 10,000. Data were then analyzed using GraphPad Prism (GraphPad Software, CA, USA), and the Kd values of the compounds were determined from competitive binding experiments as per the Cheng and Prusoff equation.

### Microscale thermophoresis (MST) assay

Human AGTR1- or AGTR2-GFP-9×His-tag fusion protein plasmids were transfected into HEK293 cells for 36 h. The proteins were homogenously purified by Ni^+^ Sepharose (Cat. #17531801, Cytiva, USA). The fusion protein was suspended in binding buffer (250 mM Tris HCl, pH 7.4, 750 mM NaCl, 50 mM MgCl_2_, 0.05% Tween-20). BSA (1% in the final binding mixture) was added to prevent adhesion of proteins to the plastic tubes and glass capillaries. Human hsa-let-7a-1/2/3 RNA plasmid was used to obtain the corresponding *in vitro*-transcribed RNA products by the MEGA shortscript High Yield Transcription Kit (Life, AM1354). The supernatant, containing the AGT1R-GFP or AGTR2-GFP fusion protein, was diluted to provide a GFP fluorescence density of 600 to 1,000 fluorescence units. The fusion protein was then divided into 16 tubes (10 μL/tube). A range of RNA solutions were prepared with a two‐fold dilution, and 10 μL of each dilution was added to the fusion protein in the 16 tubes. After mixing, the samples were siphoned into capillary tubes, and binding activity was measured by the Monolith NT.115 instrument (NanoTemper Technologies) 40. Synthetic extracellular and intracellular peptides of FICT-labelled AGTR2 at the C-terminal were suspended in binding buffer (the buffer pH value was adjusted to the isoelectric point of the peptides) and diluted to a fluorescence density of 600 to 1,000 fluorescence units. Hsa-let-7a-2 sourced *in vitro* transcript, bound to the peptide, was assayed.

### Western blot analysis

Total protein from HEK293 cells or materials eluted by ChIRP, RNA pull-down, or immunoprecipitation assay underwent 10% SDS-PAGE and were transferred to nitrocellulose membranes (Millipore, Billerica, MA, USA). The membranes were blocked with 5% fat-free milk and incubated with antibodies against AGTR1 (1:200, ab124734, Abcam, Cambridge, MA), AGTR2 (1:2000, ab92445, Abcam, Cambridge, MA), glyceraldehyde-3-phosphate dehydrogenase (GAPDH; 1:5000, ab181603, Abcam, Cambridge, MA), phosphoserine (1:1000, ab9334, Abcam, Cambridge, MA), or Rho A (1:3000, ab187027, Abcam, Cambridge, MA) at 4°C overnight. After incubating with IRDye 680 secondary antibodies at room temperature for 1 h, the bands were scanned by the Odyssey infrared imaging system (LI-COR Biosciences).

### Real-time RT-PCR

Total RNA was extracted using the miRNeasy kit (Cat:217604, QIAGEN, USA) according to the manufacturer's protocol, and quantified by nanodrop. Then, purified RNA (1 μg) was reverse transcribed to cDNA using the AMV Reverse Transcriptase Kit (Promega, Madison, WI, USA) with random primers or mature miRNA specific primers. Relative expression of pre-miRNAs and mature miRNAs was determined by TaqMan quantitative real-time RT-PCR on an ABI Prism 7,500 sequence detection system (Applied Biosystems, Foster, CA, USA) with a TaqMan Universal Master kit (Applied Biosystems). The comparative 2^-ΔΔCt^ method was used to quantify the relative miRNA expression with *GAPDH* as an endogenous control. The primers and probes used in the TaqMan quantitative real-time PCR are listed in Table S2.

### Cell treatment for AGTR2 post-receptor signaling pathway assay

Dicer^-/-^ HEK293 cells were co-transfected with AGTR2 and hsa-let-7a-2/hsa-let-7a-1/hsa-let-7a-3 plasmids for 24 h before pretreatment with losartan (5 μM) for 30 min followed by Ang II (0.2 μM) for appropriate time periods as indicated. AGTR2^-/-^, AGTR1^-/-^, Dicer^-/-^AGTR1^-/-^, and AGTR1^-/-^hsa-let-7a-2^+/-^ cells were treated similarly to Dicer^-/-^ cells, except that AGTR2^-/-^ cells were transfected without AGTR2 plasmid and Dicer^-/-^AGTR1^-/-^ and AGTR1^-/-^hsa-let-7a-2^+/-^ cells were not treated with losartan. Following treatment, the cells were used for cAMP, SHP-1 phosphatase activity and immunoprecipitation assay.

### cAMP assay

The cAMP concentration in cells was determined by a cAMP-Gi kit (Cat. #62AM9PEB, Cisbio). Cells were dispensed at 6,000 cells/well. Losartan (5 μM), forskolin (0.6 μM), and serial dilutions of Ang II (Dicer^-/-^AGTR2^-/-^ cells), or forskolin plus Ang II (AGTR1^-/-^ cells) were added and incubated for 45 min. Finally, cAMP-Cryptate and Anti cAMP-d2 were added to the solution and incubated for 1 h. Fluorescent signals were detected on a Rubystar fluorescent reader (BMG Labtech) at 665 and 620 nm.

### SHP-1 phosphatase activity assay

The phosphatase activity of SHP-1 was detected as previously reported 25. In brief, SHP-1 protein in cell lysates was incubated with anti-SHP-1 antibody (Cat. ab227503, Abcam, Cambridge, MA) at 4°C overnight and precipitated with Protein G-Agarose beads (Roche). A RediPlate 96 EnzChekR Tyrosine Phosphatase Assay Kit (Cat. R-22067) was used for the SHP-1 activity assay (Molecular Probes, Invitrogen).

### Immunoprecipitation

Interaction of AGTR2 with G_αi-2_/G_αi-3_ was determined by co-immunoprecipitation. Briefly, after transfection of HEK293 cells with AGTR2, G_αi-2_/G_αi-3_, and hsa-let-7a-2/control plasmids for 24 hours, proteins were extracted with Minute total protein extraction kit (SN002, invent Biotechnologies, Eden Prairie, UK). Then, the proteins were incubated anti-IgG (Cat. ab172730, Abcam, Cambridge, MA) or anti-AGTR2 antibody (Cat. ab92445, Abcam, Cambridge, MA) for overnight at 4 °C. Protein A/G agarose was added and incubated for an additional 4 h at 4 °C. After centrifugation, the supernatant was removed, and the immunoselections were washed three times with ice-cold Phosphate Buffer Saline. G_αi-2_/G_αi-3_ in the immunoselection was detected by western blot analysis with corresponding antibodies (anti-G_αi-2_: Cat. sc-13534AP, Santa Cruz Biotechnology, CA, USA; anti-G_αi-3_: Cat. ab173527, Abcam, Cambridge, MA).

Phosphorylation of RhoA was assayed as described 12. Primary anti-Rho A antibody (Cat. sc-418, Santa Cruz Biotechnology, CA, USA) was pre-incubated with 40 μL of protein A/G Sepharose beads at 4°C overnight. After the supernatant was removed, cell lysis buffer (RIPA lysis buffer with phosphatase inhibitors) was added at 4°C overnight with gentle rocking, and then washed three times with lysis buffer. Pellets were heated at 70°C for 10 min in 1×loading buffer. Then, SDS-PAGE was performed and immunoblotting was conducted with anti-phospho-Ser antibody (Cat. sc-81514, Sant Cruz Biotechnology) to determine RhoA serine phosphorylation. Aortic tissue lysates were examined with western blot analysis using anti-RhoA-(phospho S188) antibody (Cat. ab41435, Abcam, Cambridge, MA).

### Cell proliferation, migration, and apoptosis assays

Dicer^-/-^AGTR^-/-^ cells were co-transfected with AGTR2 plasmid and control (p-CON) / hsa-let-7a-2 (p-hsa-let-7a-2) plasmid 24 h before being treated with Ang II (0.2 μM) orthe same volume of vehicle. Twenty four hours later, cell proliferation was assessed by an EdU Cell Proliferation Assay (Flow Cytometry) Kit (Ribobio, Guangzhou, China), cell migration was examined by transwell cell culture chambers and the scratch method, and cell apoptosis was determined by an Annexin V-FITC/PI (Oncogene, San Diego, CA, USA) and TdT-mediated dUTP Nick-end labeling (TUNEL) Apoptosis Detection Kit (Beyotime Institute of Biotechnology, Shanghai, China).

### Arterial transfection and dilation

WT and AGTR1^-/-^ mice were sacrificed by a pentobarbital injection. The secondary class of mesenteric artery was dissected out and maintained in high glucose DMEM with or without hsa-let-7a-2 adenovirus and incubated in a 95% O_2_/5% CO_2_ incubator at 37°C for 12 h. For the isometric tension measurement, the arteries were cut into ring segments of approximately 2 mm in length and suspended in a wire myograph (Danish Myo Technology, Aarhus, Denmark). Then, the chamber was filled with Krebs solution (119 mM NaCl, 4.7 mM KCl, 2.5 mM CaCl_2_, 1 mM MgCl_2_, 25 mM NaHCO_3_, 1.2 mM KH_2_PO_4_, and 11 mM D-glucose) oxygenated with 95% O_2_/5% CO_2_ at 37°C (pH 7.4). The arterial rings were stretched to an optimal tension of 1 mN. After equilibrating for 30 min, the rings were contracted with 60 mM KCl, and then washed several times in warm Krebs solution. Subsequently, these arteries were incubated with phenylephrine (Phe, 10^-5^ mol/L) and isometric tension of each vessel was recorded. After reaching the maximum and steady contraction, each arterial ring was relaxed by cumulative additions of Ang II (10^-10^ to 10^-5^ mol/L). The study was conducted according to the guidelines of the Declaration of Helsinki, and approved by the Ethics Committee on Animal Study of Fuwai Hospital (FW-2021-0017).

### Statistics

Data are presented as mean ± standard deviation (SD). Differences between two groups were evaluated by unpaired Student's *t* test and among more than two groups by two-way analysis of variance (ANOVA). All statistical analyses were performed by GraphPad Prism v8.0.2. *P* < 0.05 was considered statistically significant.

## Supplementary Material

Supplementary figures and tables.

## Abbreviations

GPCR: G protein-coupled receptor
miRNA: microRNAs
Ang II: angiotensin II
AGTR2: angiotensin II type 2 receptor
pre-miRNA: precursor miRNA
TLR7: toll-like receptor 7
AGTR1: angiotensin II type 1 receptor
irCLIP: infrared-UV-C crosslinking immunoprecipitation
RIP: RNA-binding protein immunoprecipitation
RT-PCR: reverse transcription PCR
ChIRP: chromatin isolation RNA purification pull-down
MST: microscale thermophoresis
ROCK: Rho kinase
WT: wild type
HEK 293: human embryonic kidney 293
TUNEL: terminal deoxynucleotidyl transferase dUTP nick end labeling
