# Supplementary material for: Pre-miRNA Hsa-Let-7a-2: a Novel Intracellular Partner of Angiotensin II Type 2 Receptor Negatively Regulating its Signals
Source: Int J Biol Sci. 2022 May 1;18(8):3237–50. doi: 10.7150/ijbs.70455 (PMC9134915; doi:10.7150/ijbs.70455)
Supplement: Supplementary file 1 — Supplementary figures and tables. [file ijbsv18p3237s1.pdf]

Supplementary Figures and Tables

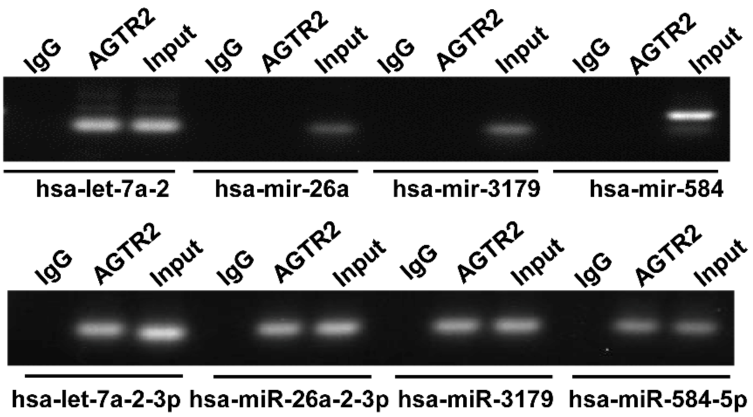

Figure S1. Identification of the miRNAs that bind AGTR2. Using RNA-binding protein immunoprecipitation, then RT-PCR with specific primers, pre-miRNAs and mature miRNAs were detected.

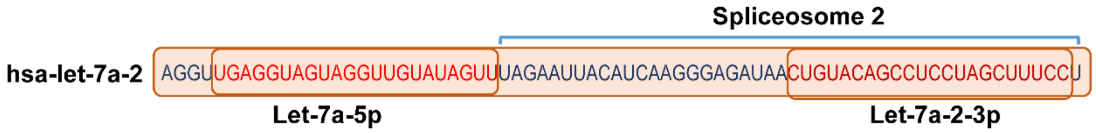

Figure S2. The sequence of hsa-let-7a-2 and its spliceosomes.

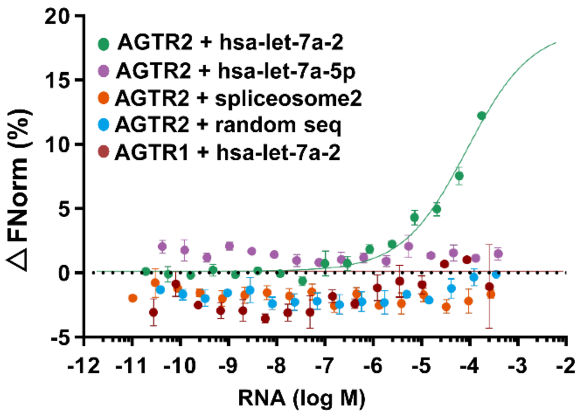

Figure S3. Identification of hsa-let-7a-2 and other spliceosomes by the microscale thermophoresis (MST) assay. With purified AGTR1-GPF or AGTR2-GFP fusion protein as a receptor and synthetic RNAs as ligands, the interaction of RNA with the receptor was detected by MST.

hsa-let-7a-1: UGGGAUGAGGUAGUAGGUUGUAUAGUUUUAGGGUCACACCCACCACUGGGAGAUAACUAUACAAUCUACUGUCUUUCCUA

hsa-let-7a-2: ...AGGUUGAGGUAGUAGGUUGUAUAGUUU............AGAAUUACAUCAAGGGAGAUAACUGUACAGCCUCCUAGCUUUCCU...

hsa-let-7a-3: .... GGGUGAGGUAGUAGGUUGUAUAGUUU......GGGGCUCUGCCCUGCUAUGGGAUAACUAUACAAUCUACUGUCUUUCCU...

Figure S4. Sequence analysis of hsa-let-7a-1/2/3. Yellow represents the same sequences; red font represents the different sequence of hsa-let-7a-2 with hsa-let-7a-1/3.

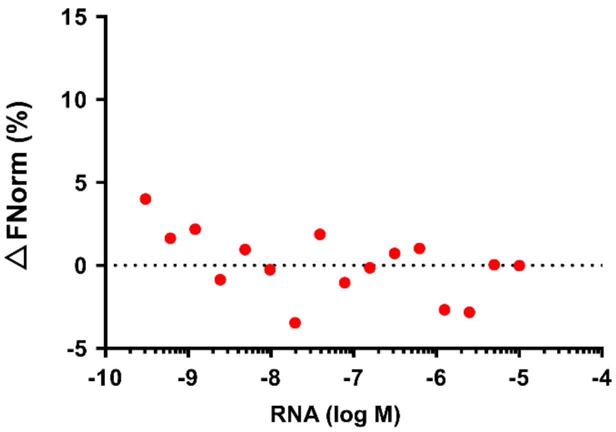

Figure S5. **hsa-let-7a-2 did not bind to boiled AGTR2 as determined by microscale thermophoresis.**

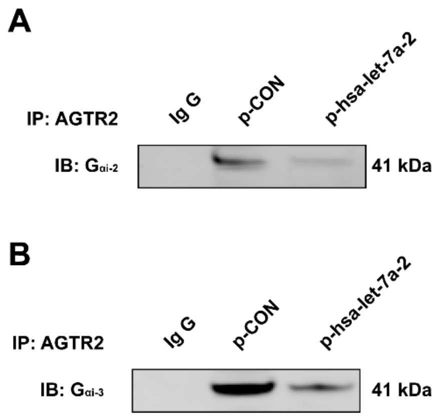

Figure S6: Hsa-let-7a-2 competes with G_{ai-2}/G_{ai-3} in combination of AGTR2. A. Hsa-let-7a-2 competes with Ga_{i-2} in combination of AGTR2. B. Hsa-let-7a-2 competes with G_{ai-3} in combination of AGTR2.

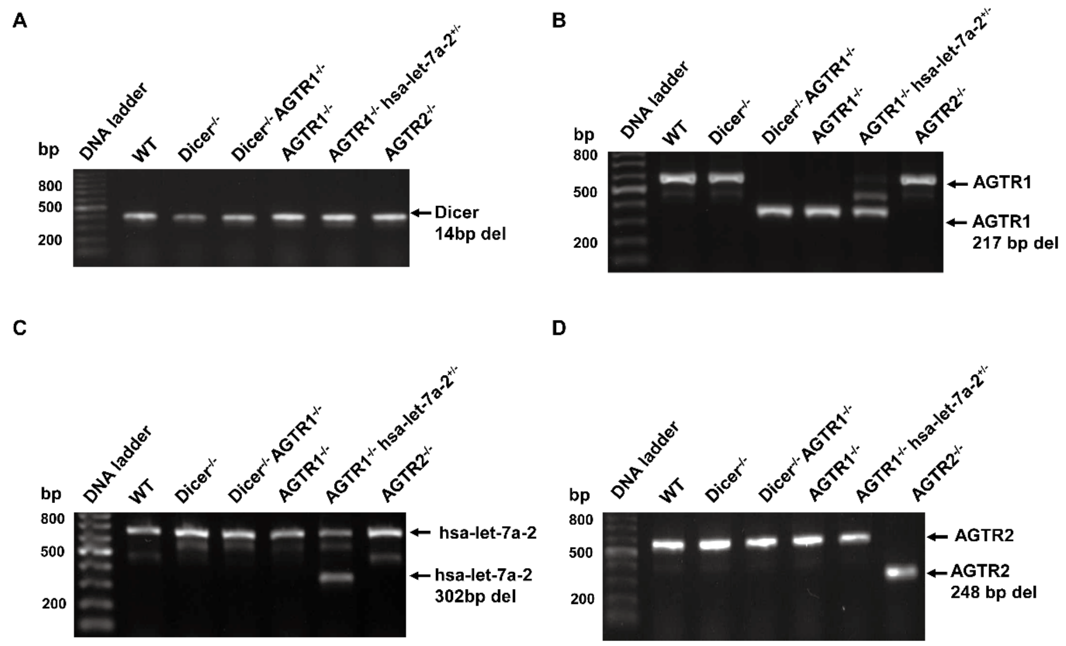

Figure S7. Verification of gene knockout by PCR. Dicer (A), AGTR1 (B), hsa-let-7a-2 (C) AGTR2 (D), Dicer and AGTR1 or AGTR1 and MIRLET7A2 double-knockout cells were prepared by CRISPR-Cas9 assay and identified by PCR.

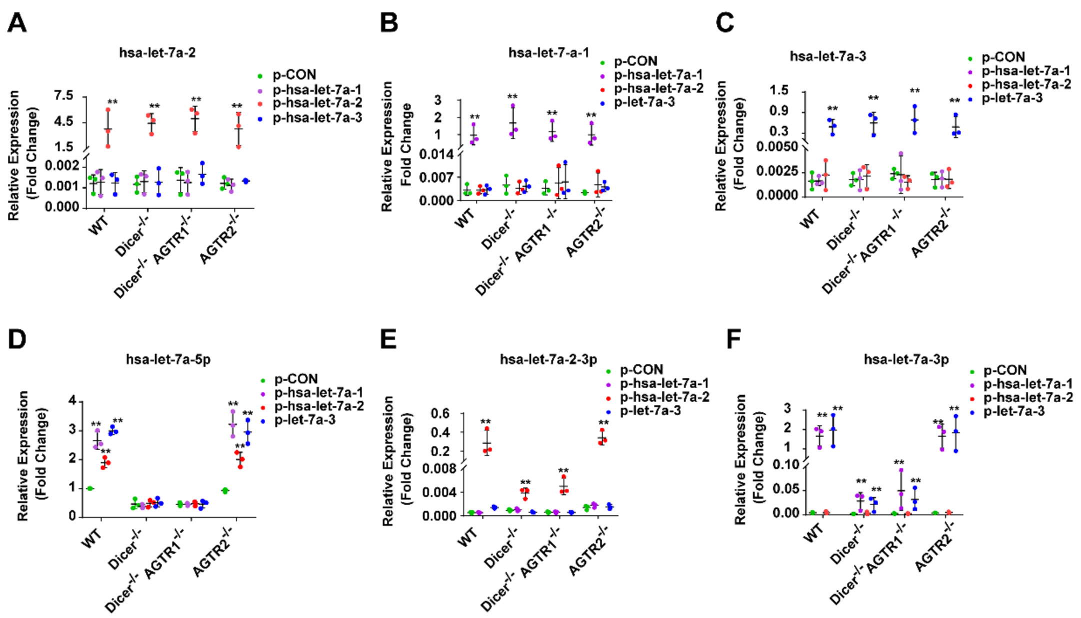

Figure S8. Expression of pre-miRNAs and mature miRNAs in cells transfected with pre-miRNA plasmids. (A) Hsa-let-7a-2 was significantly upregulated in wild type (WT), Dicer$^{-/-}$, Dicer$^{-/-}$AGTR1$^{-/-}$ and AGTR2$^{-/-}$ HEK-293 cells transfected with

hsa-let-7a-2 plasmid (p-hsa-let-7a-2); (B) Hsa-let-7a-1 was significantly upregulated in WT, Dicer$^{-/-}$, Dicer$^{-/-}$AGTR1$^{-/-}$ and AGTR2$^{-/-}$ HEK-293 cells transfected with hsa-let-7a-1 plasmid (p-hsa-let-7a-1); (C) Hsa-let-7a-3 was significantly upregulated in WT, Dicer$^{-/-}$, Dicer$^{-/-}$AGTR1$^{-/-}$ and AGTR2$^{-/-}$ HEK-293 cells transfected with hsa-let-7a-3 plasmid (p-hsa-let-7a-3); (D) Hsa-let-7a-5p was significantly upregulated in WT and AGTR2$^{-/-}$ HEK-293 cells but not Dicer$^{-/-}$or Dicer$^{-/-}$AGTR1$^{-/-}$ cells transfected with p-hsa-let-7a-1/2/3; (E) Hsa-let-7a-2-3p was significantly upregulated in WT and AGTR2$^{-/-}$ HEK-293 cells but mildly upregulated in Dicer$^{-/-}$or Dicer$^{-/-}$AGTR1$^{-/-}$ cells transfected with p-hsa-let-7a-2; (F) Hsa-let-7a-3p was significantly upregulated in WT and AGTR2$^{-/-}$ HEK-293 cells but mildly upregulated in Dicer$^{-/-}$ and Dicer$^{-/-}$AGTR1$^{-/-}$ cells transfected with p-hsa-let-7a-1/3. **P<0.01, Data are expressed as mean ± SD. N=3.

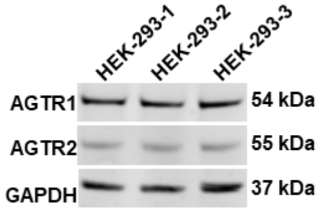

Figure S9. Confirmation of AGTR1 and AGTR2 protein expression in HEK-293 cells.

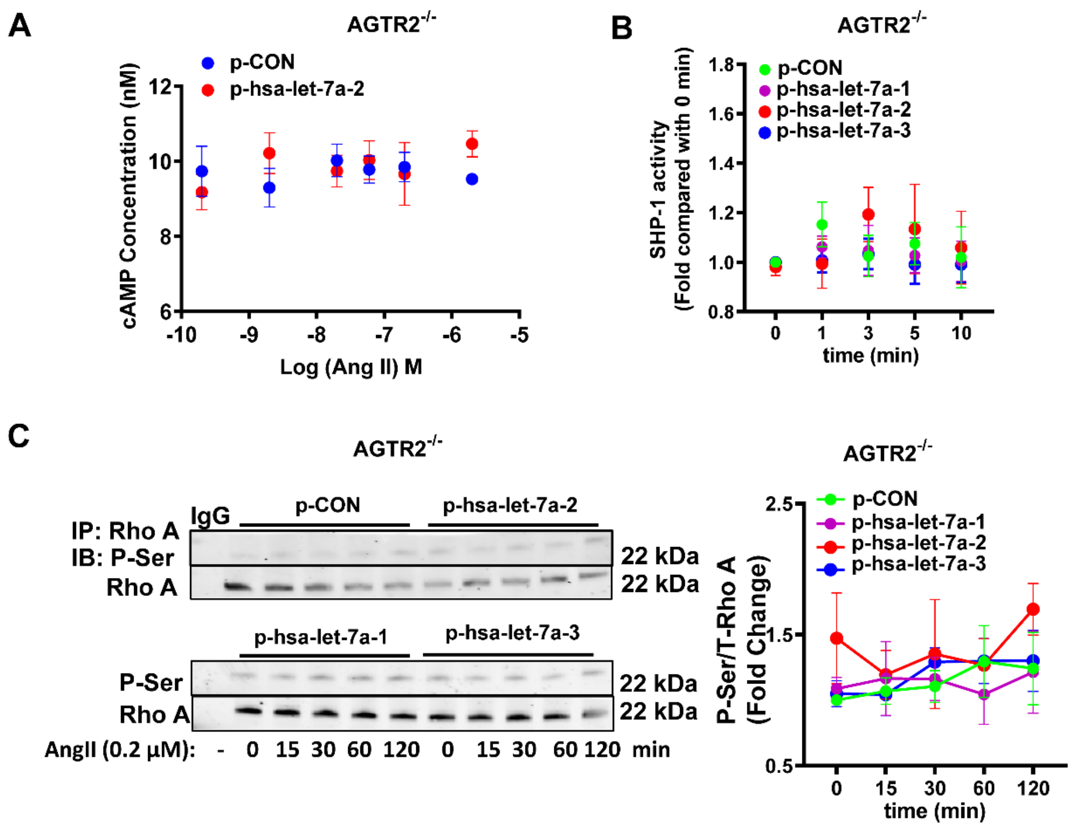

Figure S10. AGTR2 signaling changes in AGTR2-knockout cells (AGTR2$^{-/-}$). (A) cAMP changes with overexpression of hsa-let-7a-2 stimulated with gradient concentration of AngII. (B) Effect of overexpression of hsa-let-7a-2 or its homogenous hsa-let-7a-1&3 on SHP-1 activity in AGTR2$^{-/-}$ cells. (C) Effect of elevated hsa-let-7a-2 or hsa-let-7a-1/3 level on RhoA phosphorylation at serine sites in AGTR2$^{-/-}$ cells. Data are expressed as mean ± SD. N=3-5.

Table S1. sgRNAs sequences for AGTR1, AGTR2, DICER, and MIRLET7A2

Gene name	sgRNA number	sequence
**AGTR1**	AGTR1-sgRNA1	CAAGATGATTGTCCCAAAGC
	AGTR1-sgRNA2	ACACAGCTATGGAATACCGC
**AGTR2**	AGTR2-sgRNA1	AAGCCCGAAGTGAAGACCGC
	AGTR2-sgRNA2	GAATAATAGGTTGCCCATAG
**DICER**	DICER-sgRNA1	TGCTTCCTCACCAATGGGTC
	DICER-sgRNA2	TCGTCTGTTTAAACACTGGC
	DICER-sgRNA3	GGTTGCTCAACAAGTGTCAG
	DICER-sgRNA4	TGCTGAAACTGCAACTGACC
	DICER-sgRNA5	TGTGAGATTGTGGTGGATTG
	DICER-sgRNA6	TGGTGTGCAGATAAAGTAGC
	DICER-sgRNA7	ATGAGCAAGAGGAGCTGCAC
	DICER-sgRNA8	TGGCAAACAAGATCCAGAGC
**MIRLET7A2**	MIRLET7A2-sgRNA1	CTTCTTGAACTATTTGTGCA
	MIRLET7A2-sgRNA2	GTTGTTTAGTGCAAGACCCA

Table S2. Primers and probes for RT-PCR and qPCR

Gene name	Primer for reverse transcription	qPCR primer-forward	qPCR primer-reverse	probe
**hsa-let-7a-2**	Random primer	GCAGGAAAGCTAGGAGGCTGT	CGAGGTTGAGGTAGTAGGTTGTATAGTTTAG	CAGTTATCTCCCTTGATGTAA
**Let-7a-5p**	GTCGTATCCAGTGCGTGTCGTGGAGTCGGCAATTGCACTGGATACGACAACTAT	GCCGGCTGAGGTAGTAGGTTG	GCGTGTCGTGGAGTCGG	TCGTATCCAGTGCAATT
**Let-7a-2-3p**	GTCGTATCCAGTGCGTGTCGTGGAGTCGGCAATTGCACTGGATACGACGGAAAG	GCCGTTCTGTACAGCCTCCTA	GCGTGTCGTGGAGTCGG	TCGTATCCAGTGCAATT
**hsa-mir-26a**	Random primer	TCAAGTAATCCAGGATAGGCTGTG	TCCCCGTGCAAGTAACCAA	TCCCAATGGGCCTATT
**mir-26a-2-3p**	GTCGTATCCAGTGCAGGGTCCGAGGTATTCGCACTGGATACGACGAAACA	CAGTGCAGGGTCCGAGGTA	GCCGGGCCTATTCTTGATTACT	TCGCACTGGATACGAC
**hsa-mir-584**	Random primer	TTATGGTTTGCCTGGGACTGA	GCTTCAGGGAGACCAACCAG	TCAGTTCCAGGCCAAC
**mir-584-5p**	GTCGTATCCAGTGCAGGGTCCGAGGTATTCGCACTGGATACGACCTCAGT	CAGTGCAGGGTCCGAGGTA	GCGTTATGGTTTGCCTGGG	TCGCACTGGATACGAC
**hsa-mir-3179**	Random primer	GGATCACAGACGTTTAAATTACACTCC	AGACGTTTAAATTTCACCCCTTCTACT	TCTGCTGTGCCTTACAG
**mir-3179**	GTGGTACCAGTGCAGGGTCCGAGGTATTCGCACTGGATACGACACGTTT	CAGTGCAGGGTCCGAGGTA	GCCGAGAAGGGGTGAAATTT	TCGCACTGGATACGAC
**GAPDH**	Random primer	GGGCTGCTTTTAACTCTGGTAAAG	CCATGGGTGGAATCATATTGG	CCTCAACTACATGGTTTAC
